# Intake of Erythrocytes Required for Reproductive Development of Female *Schistosoma japonicum*

**DOI:** 10.1371/journal.pone.0126822

**Published:** 2015-05-15

**Authors:** Jipeng Wang, Shuqi Wang, Xiufeng Liu, Bin Xu, Riyi Chai, Pan Zhou, Chuan Ju, Jun Sun, Paul J. Brindley, Wei Hu

**Affiliations:** 1 Department of Microbiology and Microbial Engineering, School of Life Sciences, Fudan University, Shanghai, China; 2 Key Laboratory of Parasite and Vector Biology of Ministry of Public Health, Institute of Parasitic Diseases, Chinese Center for Disease Control and Prevention, Shanghai, China; 3 Institute for Infectious Diseases and Vaccine development, Tongji University School of Medicine, Shanghai, China; 4 Department of Microbiology, Immunology & Tropical Medicine, and Research Center for the Neglected Diseases of Poverty, School of Medicine & Health Sciences, George Washington University, Washington, DC, United States of America; Queensland Institute of Medical Research, AUSTRALIA

## Abstract

The reproductive development and maturation of female schistosomes are crucial since their released eggs are responsible for the host immunopathology and transmission of schistosomiasis. However, little is known about the nutrients required by female *Schistosoma japonicum* during its sexual maturation. We evaluated the promoting effect of several nutrients (calf serum, red blood cells (RBCs), ATP and hypoxanthine) on the reproductive development of pre-adult females at 18 days post infection (dpi) from mixed infections and at 50 dpi from unisexual infections of laboratory mice in basic medium RPMI-1640. We found RBCs, rather than other nutrients, promoted the female sexual maturation and egg production with significant morphological changes. In 27% of females (18 dpi) from mixed infections that paired with males *in vitro* on day 14, vitelline glands could be positively stained by Fast Blue B; and in 35% of females (50 dpi) from unisexual infections on day 21, mature vitelline cells were observed. Infertile eggs were detected among both groups. To analyze which component of mouse RBCs possesses the stimulating effect, RBCs were fractionated and included in media. However, the RBC fractions failed to stimulate development of the female reproductive organs. In addition, bovine hemoglobin hydrolysate, digested by neutral protease, was found to exhibit the promoting activity instead of untreated bovine hemoglobin. The other protein hydrolysate, lactalbumin hydrolysate, exhibited a similar effect with bovine hemoglobin hydrolysate. Using quantitative RT-PCR, we found the expression levels of four reproduction-related genes were significantly stimulated by RBCs. These data indicate that RBCs provide essential nutrients for the sexual maturation of female *S*. *japonicum *and that the protein component of RBCs appeared to constitute the key nutrient. These findings would improve laboratory culture of pre-adult schistosomes to adult worms in medium with well-defined components, which is important to investigate the function of genes related to female sexual maturation.

## Introduction

Schistosomiasis is a worldwide distributed disease infecting over 200 million people and threatening nearly 800 million [1]. Despite more than 50 years of exerted effort to control *Schistosomiasis japonica* in China, this infectious disease remains prevalent in seven provinces including Hubei, Sichuan and Anhui [2]. The pathogenicity of schistosomes is mainly due to host immune responses to the eggs produced by mature female worms [3]. The reproductive development of female schistosomes is male-dependent. Male and female *Schistosoma japonicum* develop independently until they form pairs about 18 days post infection (dpi) [4]. Once the worm pairs are *in copula*, the gonads of the female develop to maturation for egg production [5–7]. The maturation of vitellaria and ovaries fails to commence in virgin female schistosomes [8, 9]. The mechanism of this male-dependent female sexual maturation remains to be fully elucidated although the phenomenon is the focus of substantial interest [10]. In addition to continuous pairing with the male, the maturation of female schistosomes requires a considerable quantity of nutrients, from ingested blood, to support maturation and the release of several thousand eggs daily from each female *S*. *japonicum* for many months or years [11]. Therefore, the knowledge of the essential nutritional demand for the reproductive development in schistosomes is of importance to optimize culture conditions that, in turn, can be expected benefit to the *in vitro* studies of the male-female interplay.

Cultivating schistosomes outside their hosts has advantages over that *in vivo* since the culture medium is more stable and less complex, which provides a reliable, tractable platform to study the physiology and nutritional needs of the schistosomes. By mimicking the host nutritional environment, a number of culture media for discrete developmental stages of the schistosomes have been reported [12]. *S*. *japonicum* and *Schistosoma mansoni* can be cultured from cercariae to paired adults in rich medium 841 and 169, respectively. These are media that include nutrients required for growth, development and reproduction of schistosomes, such as glucose, lactalbumin hydrolysate, hormones, erythrocytes, etc. [13–16]. Although the development of schistosomes in the mammal can be approximately mimicked during culture in rich media, the essential nutritional needs of schistosomes during the sexual maturation of the female remain unclear. By contrast, basic culture media, such as RPMI-1640 and DMEM, are widely used with defined modifications for genetic manipulation and drug screening on schistosomula and adults *in vitro* [12, 17, 18]. Less complex, yet well defined, media can be expected to facilitate the investigation of the nutritional requirements for schistosomes during their sexual maturation *in vitro*.

Mammalian blood is a naturally rich medium. Many nutrients in the plasma, such as glucose [19], fatty acids [20] and albumin [21], are utilized by schistosomes. *In vivo* and *in vitro* studies have demonstrated that some host nutrients exhibited a stimulating effect on the growth of schistosomes. Erythrocytes, the principal solid component in blood, are ingested by schistosomes and the amino acids of hemoglobin released from erythrocytes are absorbed through the gut [22, 23]. Red blood cells (RBCs) and fractions of RBCs positively influence the growth of the schistosomula of *S*. *mansoni in vitro* [24, 25]. In addition, RNA interference of the cathepsin B and D, proteinases that are involved in hemoglobin digestion, led to the early growth retardation of *S*. *mansoni* [26, 27]. The energy requirements for schistosomes evolve during development [28] and certain as yet undefined nutrients might be involved in the process of reproductive development. Paired adult females intake several fold more RBCs than virgins from unisexual infections [29]. Neutral lipid is not found in virgins, but lipid drops are formed in the vitellaria of adult females [30]. ATP and hypoxanthine are reported to improve the oviposition of adult female *S*. *japonicum* during *in vitro* cultivation [31]. However, the promoting effect of these nutrients on the development of the reproductive system has not been investigated.

To investigate the nutritional requirement during the development of schistosomes from pre-adult to adult stage, here we cultured paired pre-adult female *S*. *japonicum* in basic medium RPMI-1640 and examined the morphological changes of the reproductive system. RBCs, rather than other nutrients, promoted the sexual maturation of immature female schistosomes. We evaluated the effect of fractions of RBCs to determine which part contributes to the promoting effect. Notably, protease digested hemoglobin had an effect that was the same as that of RBCs, and superior to untreated hemoglobin, on the development of feminine reproductive organs. Besides, we found the expression of the female reproduction-related genes were highly influenced by RBCs using RT-PCR analysis. These findings indicate that erythrocytes are a prerequisite for the sexual maturation of the female of *S*. *japonicum* besides being in copula with males, for it provided essential nutrients for reproductive development and that the protein component was the key nutrient.

## Materials and Methods

### Parasites

Cercariae of *S*. *japonicum* (Anhui isolate), released freshly from infected *Oncomelania hupensis* snails, were provided by the pathogen biology laboratory of the National Institute of Parasitic Diseases, Chinese Center for Diseases Control and Prevention, Shanghai. For mixed infections, cercariae released from multiple infected *O*. *hupensis* snails were used. Each mouse was infected with 150–200 cercariae dispensed onto the shaved abdomen of the mouse. Pre-adult worms were obtained 18 days later by hepatic-portal perfusion [32]. According to the results of a previous experiment with mono-miracidial infections [33], the yield ratio of mono-miracidia infected snails was only 4%. Therefore, we adjusted the strategy employed here by limited-miracidial infection. The negative snails and the miracidia were incubated with a certain proportion (snails: miracidia = 1: 2~4) for infections. Then the cercariae released from one limited-miracidial infected snail were used to infect each mouse. 80–100 cercariae were administered to each mouse. Worms were recovered at 50 dpi, as above. In this infection, only the unisexual worms were used for further study.

### Animals and ethics

Female Kunming strain mice (18–20 g) were purchased from the Shanghai Animal Center, Chinese Academy of Sciences (Shanghai, China). The mice were housed 5 per cage and kept in the room controlled with a constant temperature (22±2°C) and humidity (60–80%) under 12 h light/dark cycle. The provided food and water were freely available and monitored daily. The animal work was approved by the Ethics Committee of the National Institute of Parasitic Diseases, Chinese Center for Disease Control and Prevention in Shanghai, China (Ref No: 20100525–1). The use of mice in these experiments conformed with the guidelines for the Care and Use of Laboratory Animals of the Ministry of Science and Technology of People's Republic of China ([2006]398). All animals were euthanized by cervical dislocation and then dissected to harvest the worms.

### Cultivation *in vitro*

Basic culture medium RPMI-1640 (Gibco, USA) containing 20% calf serum (Zhejiang Tianhang Biological Technology Co., Ltd, Zhejiang, China), 100 IU/ml penicillin sodium, 100 IU/ml streptomycin, and 0.25 μg/ml amphotericin B (Hyclone, USA), was used to maintain schistosomes *in vitro*. Worms were cultured in a 12-well plate with 4 ml culture medium per well. To culture pre-adult worms (18 dpi) from mixed infections, 10–12 females and 10–12 males were placed in each well. To culture worms from unisexual infections, five females and five males were placed in each well. Plates were incubated at 37°C containing 5% CO_2_ and the media were refreshed every 2–3 days.

### RBCs and fractions

Approximately 5 ml whole blood obtained from female Kunming strain laboratory mice was dispensed into tubes containing anticoagulant. The tubes were centrifuged at 600 *g* for 5 min at 4°C. Supernatant blood serum and the white cell (buffy coat) were removed, after which 5 ml cold sterile saline was added into the tube to gently wash the packed red blood cells (RBCs). The centrifugation and washing procedure was repeated at least three times until the supernatant was completely clear. The supernatant was removed and the packed RBCs were transferred to a sterile tube. An equal volume of RPMI-1640 without serum was added to the tube and the RBCs could be stored at 4°C for up to two weeks.

The mouse RBCs in RPMI-1640 were disrupted by ultrasonic waves under 100 W for 20 times with a pattern of 3 s “on” and 3 s “off”. The lysate of RBCs was centrifuged at 1,500 *g* for 10 min at 4°C. Supernatant was transferred to a sterile tube and stored at 4°C. White ghosts (WGs) and pink ghosts (PGs) were prepared according to Basch et al [25].

### Additives

ATP (Amresco, USA), hypoxanthine (Sigma, USA), lactalbumin hydrolysate (Difco, USA), hemoglobin from bovine blood (Sigma) and bovine hemoglobin hydrolysate were added to the basic medium and the effects as nutrients were evaluated. The additives were dissolved in the basic medium and sterilized through 0.22 μm membrane filters and their final concentrations were 0.2 mg/ml, 5×10^-7^ M, 2 mg/ml, 2 mg/ml and 2 mg/ml, respectively.

To produce bovine hemoglobin hydrolysate, 2 g crystalline bovine hemoglobin was dissolved in 45 ml deionized water. Subsequently, 0.133 g neutral protease (Solarbio, China) was added to the solution to reach a desired concentration (4,000 U/g) for hydrolysis. Digestion proceeded at pH 7.5, 45°C for 10 h. The pH of solution was maintained at 7.5 by addition of 2 M NaOH, as needed, every 2 h. The reaction was stopped by incubation at 90°C for 30 min. Tubes were centrifuged at 4°C at 1,500 *g* for 15 min. The supernatant was transferred into 50 ml tubes and snap frozen in liquid nitrogen. Hemoglobin hydrolysate was vacuum-dried overnight in a vacuum concentrator (Labconco, USA). Pellets were collected and stored at room temperature. The proteolysis of the bovine hemoglobin was analyzed on SDS-PAGE gels (12%) stained with Coomassie Blue.

### Light microscopy

The phenolic substances in mature vitelline cells can be positively stained with Fast Red B [34]. In the present study, Fast Red B was substituted by Fast Blue B (Sinopharm Chemical Reagent Co., Ltd, China), a related diazonium salt which exhibits a similar staining profile [35]. Female worms were fixed in 70% ethanol for at least 24 h, and then stained with filtered 1% Fast Blue B solution for 1 min. The worms were dehydrated through an ethanol gradient and mounted in neutral balsam (Sinopharm Chemical Reagent Co., Ltd, China). The staining of vitelline cells was evaluated by light microscopy using a compound binocular microscope (Nikon NI-SS, Japan) under 40×.

### Confocal laser scanning microscopy (CLSM)

For morphological analysis of the reproductive organs, female worms were fixed in AFA (alcohol 95%, formalin 3%, glacial acetic acid 2%) for at least 24 h, then stained with hydrochloric carmine for 30 min, and destained in acidic 70% ethanol until the worm turned light pink. After sequential dehydration in 70%, 90%, and 100% ethanol, for 10 min respectively, worms were mounted on glass slides with neutral balsam [36, 37]. Images were collected with a Leica TCS-SP5 Spectral Laser Scanning Confocal Microscope (Leica, Germany), using a 488-nm He/Ne laser.

### Quantitative RT-PCR

Total RNA samples were isolated from female worms from mixed infections cultured for 1, 5, 10 and 15 days with and without RBCs using Trizol reagent (Invitrogen, USA). One μg total RNA from each sample was synthesized to cDNA using PrimeScript RT reagent Kit with gDNA Eraser (Takara, Japan). The expression of four *S*. *japonicum* genes (eggshell protein 1, chorion, tyrosinase 2, ferritin 1, below) [38, 39] were detected on Mx3000P platform (Agilent, USA) using SYBR Premix Ex Taq II (Tli RNaseH Plus) (Takara, Japan) with the gene encoding PSMD (26S proteasome non-ATPase) [40] as the reference calibrator. All reactions were performed in triplicate at a final volume of 25 μl with the following PCR conditions: 1) initial activation at 95°C for 30 s; 2) 40 cycles of 95°C for 5 s and 60°C for 20 s; 3) 95°C for 15 s, 60°C for 30 s and 95°C for 15 s. The primers of these target genes were designed on Primer Premier 5.0. The primer sequences for eggshell protein 1 (FN316818.1) are forward *5’-TGGTGGACCCGACTTTTATG-3’* and reverse *5’-CCTCCTTTGCCTCCGTTA-3’*; for chorion (AB017095.1) are forward 5’-GACTACAACTCCGACTACAC-3’ and reverse *5’-CCTCTGACATCTAAACGACCA-3’*; for tyrosinase 2 (AY812904.1) are forward *5’- CTTTTCCAACAACCGATAACCTCT-3’* and reverse *5’-AAACCCGCGACATTTACCC-3’*; for ferritin 1 (AF040385.1) are forward primer *5’-AGTCCGAAGAAGAGCGAC-3’* and reverse *5’-CTCACCAACGGCAACTAAATC-3’*. The primers of *PSMD* are identical with those reported by Liu [40], which are forward *5’- CCTCACCAACAATTTCCACATCT* and reverse *5’-GATCACTTATAGCCTTGCGAACAT-3’*.

### Statistical analysis

All data are expressed as mean ± SEM for 3 independent experiments and analyzed by Student’s t-test. If the *p*-value is below 0.05, the difference between two groups was considered statistically significant.

## Results

### Medium supplemented with RBCs supported reproductive development of pre-adult female *S*. *japonicum*

#### Female worms from mixed infections

**Fig 1:**
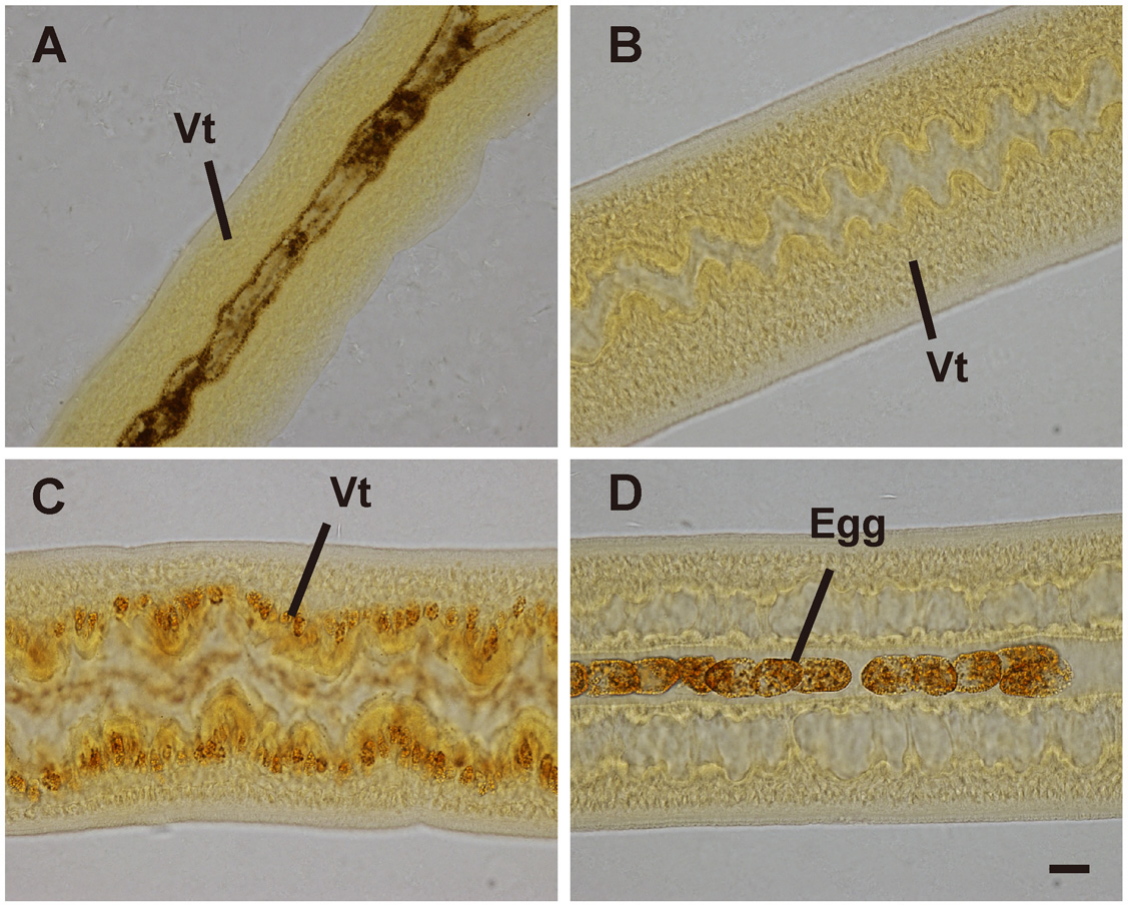
Fast Blue B staining of whole-mounted female *S*. *japonicum* from mixed infections cultured *in vitro*. (A) Females of 18 dpi. (B) Females cultured for 14 days in medium RPMI-1640 without RBCs. (C, D) Females cultured for 14 days in medium RPMI-1640 with RBCs. Vt, vitelline cells. Scale bar, 20 μm.

**Fig 2:**
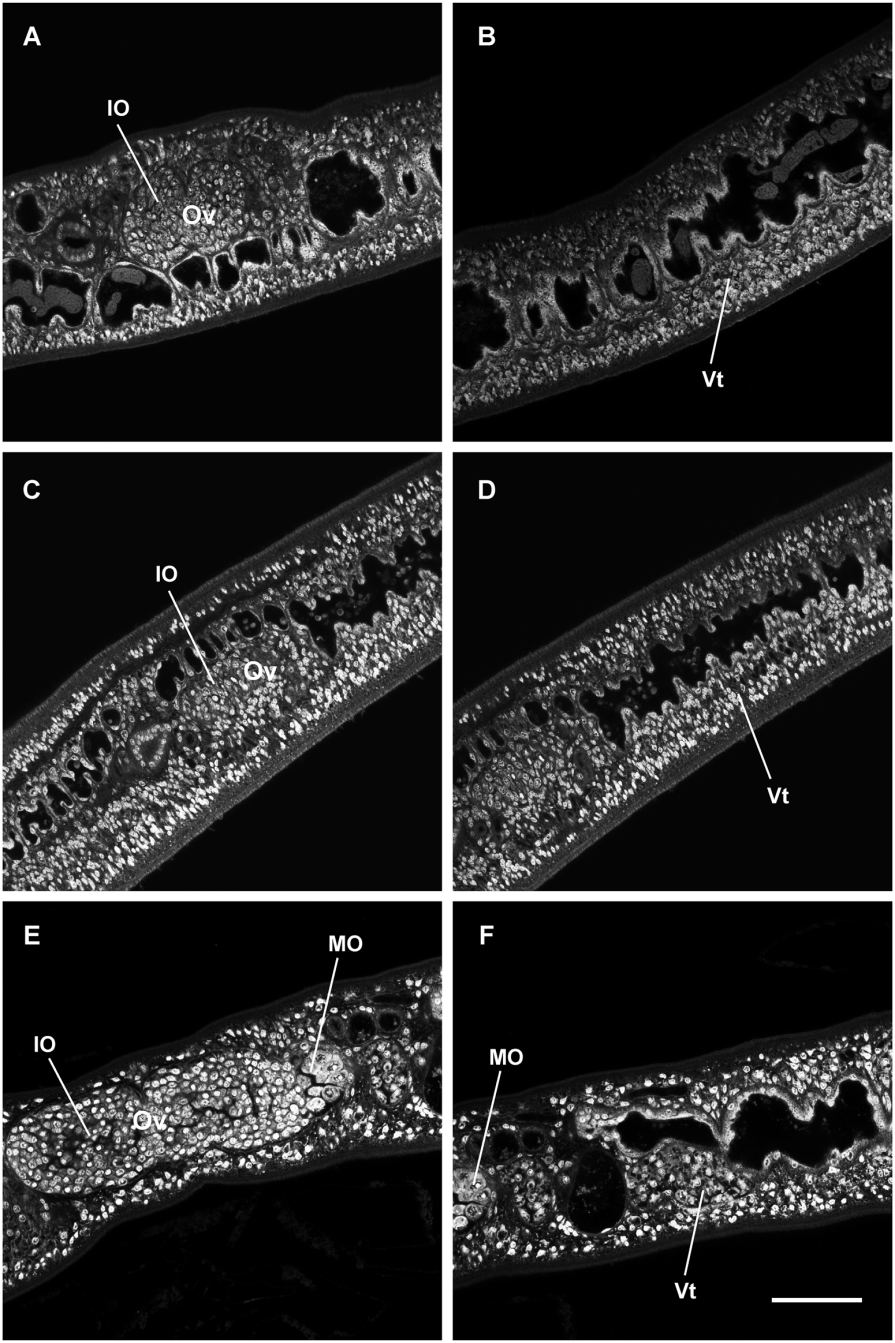
Confocal laser scanning microscopy images of female *S*. *japonicum* from mixed infections cultured *in vitro*. Females of 18 dpi before cultivation (A, B), females cultured for 14 days in medium RPMI-1640 without RBCs (C, D) or with RBCs (E, F). Ov, ovary; IO, immature oocyte; MO, mature oocyte; Vt, vitelline cells. (A) Ovary full of immature oocytes. (B) Undeveloped vitelline gland. (C) Ovary with immature oocytes. (D) Immature vitelline cells. (E) Enlarged ovary with mature oocytes. (F) Mature vitelline cells. Scale bar, 50 μm.

Female worms (18 dpi) from mixed infections were likely to be primed to commence reproductive system development since they had begun to pair with males *in vivo*, but no maturation of vitelline cells or oocytes was yet evident (Fig 1A, Fig 2A and 2B). When cultured overnight in the basic medium RPMI-1640, most pre-adult female and male worms (18 dpi) formed pairs. However, the vitelline glands of paired females cultured in basic medium failed to stain with Fast Blue B, even after extended culture for 120 days (Table 1). This indicated that whereas basic medium supported the survival of worms, it was insufficient to support development of the gonads. To augment the availability of lipids in the cultures, concentration of calf serum supplementing the medium was increased to 35%, 50% and 100%. After 14 days, the vitelline cells of paired females remained undeveloped even though the activities of the worms in the medium with high concentrations of serum appeared similar to those in the basic medium (Table 1,Fig 1B). Likewise, the addition of the spawning promoting substances (ATP, hypoxanthine) failed to promote the maturation of vitellaria (Table 1). When mouse erythrocytes (RBCs) (1% by volume) were included, Fast Blue B stained vitelline cells (orange red, color in appearance) were observed in 26.9% of paired females at 14 days after cultivation (Table 1); well-shaped eggs were also seen in the uterus of some worms (Fig 1C and 1D).

**Table 1:** The percentage of female *S*. *japonicum* exhibiting vitelline cell development in media with different additives.

Worms for cultivation	Culture medium	Incubation time (day)	Positive with Fast Blue B/ females of each experiment	Percentage of positive (range per experiment)
Worms from mixed infections (18 dpi)	Basic medium	14–120	0/33; 0/24; 0/15; 0/7; 0/22; 0/17; 0/45	0
	Basic medium with (35%, 50%, 100%) calf serum	14	0/34; 0/30; 0/32; 0/24	0
	Basic medium + 0.2mg/ml ATP	14	0/30; 0/31; 0/40	0
	Basic medium + 5×10–7 M hypoxanthine	14	0/21; 0/25; 0/13; 0/34	0
	Basic medium + RBCs (1%)	14	11/24; 6/22; 5/24; 2/8; 3/20; 5/21	26.9 (15–45.8)
Worms from unisexual infections (50 dpi)	Basic medium	14	0/9; 0/9; 0/12	0
	Basic medium + RBCs (1%)	14	0/5; 0/9; 0/11	0
	Basic medium	21	0/8; 0/7; 0/12	0
	Basic medium + RBCs (1%)	21	8/24; 4/15; 5/9	35.4 (26.7–55.6)

CLSM was deployed to examine the morphological changes of the female reproductive organs promoted by RBCs *in vitro*. Pre-adult females (18 dpi) exhibited an incipient ovary containing numerous oogonia (Fig 2A). The vitelline glands appeared undeveloped and mature vitelline cells were not evident (Fig 2B). Following culture in basic medium for 14 days, changes were not obvious in the appearance of ovaries and vitelline glands (Fig 2C and 2D). By contrast, the reproductive system developed significantly when cultured in erythrocyte-supplemented medium. Developing oocytes were present in ovaries. Ovaries elongated and were divided into two regions that contained mature and immature oocytes, respectively (Fig 2E). The vitelline gland was relatively well-developed, but contained fewer mature vitelline cells than females *in vivo* (Fig 2F) [41].

### Virgin females

**Fig 3:**
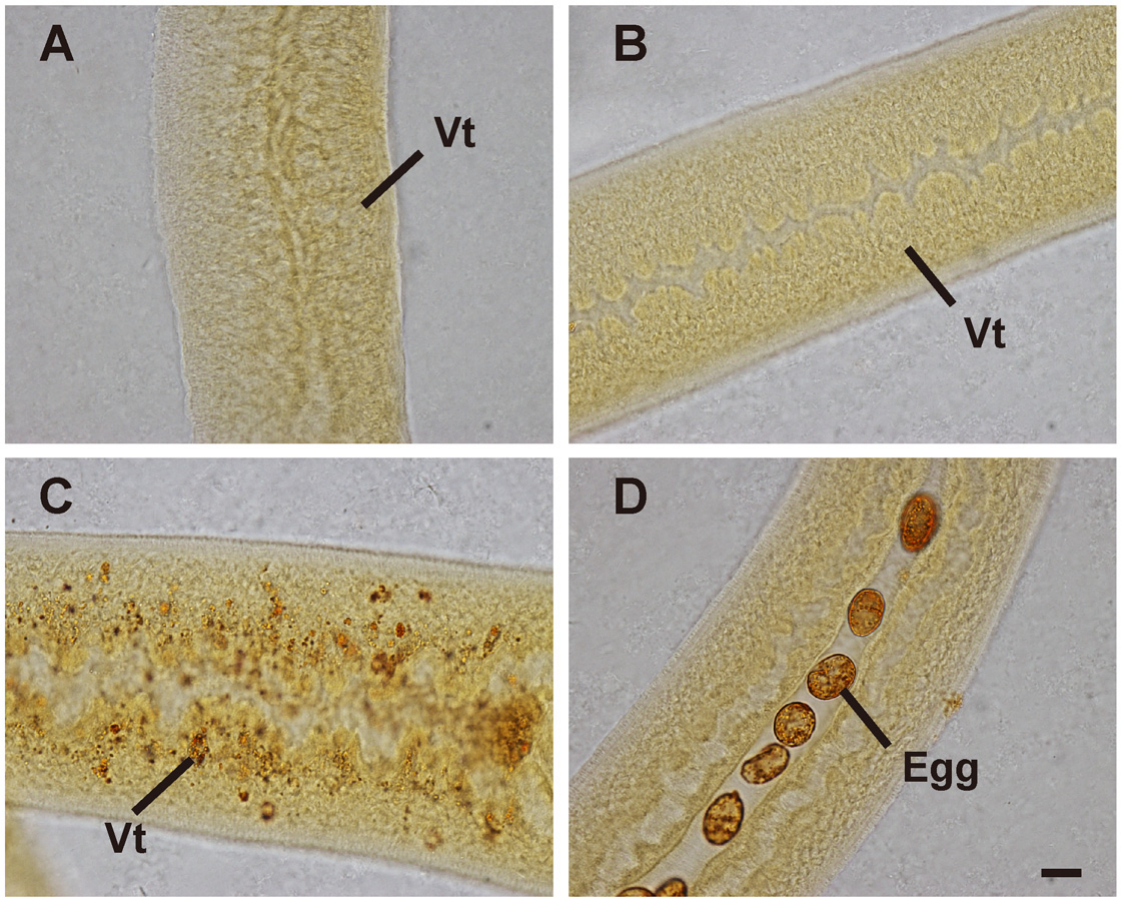
Fast Blue B staining of whole-mounted female *S*. *japonicum* from unisexual infections cultured *in vitro*. (A) Females of 50 dpi. (B) Females cultured for 21 days in medium RPMI-1640 without RBCs. (C, D) Females cultured for 21 days in medium RPMI-1640 with RBCs. Vt, vitelline cells. Scale bar, 20 μm.

**Fig 4:**
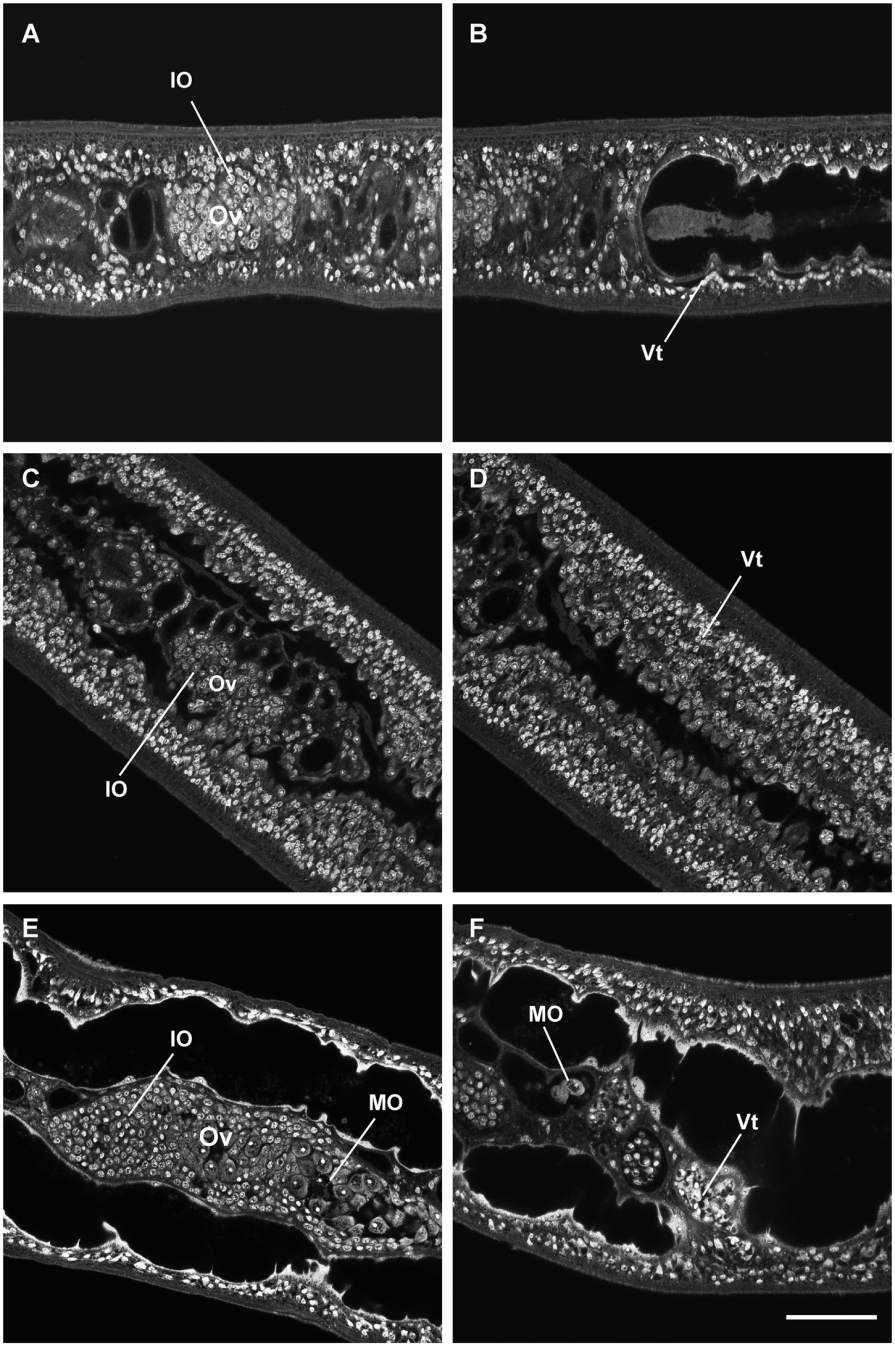
Confocal laser scanning microcopy images of female *S*. *japonicum* from unisexual infections cultured *in vitro*. Females before cultivation (A, B), females cultured for 21 days in medium RPMI-1640 without RBCs (C, D) or with RBCs (E, F). Ov, ovary; IO, immature oocyte; MO, mature oocyte; Vt, vitelline cells. (A) Ovary containing numerous immature oocytes. (B) Undeveloped vitelline gland. (C) Ovary with immature oocytes. (D) Immature vitelline cells. (E) Enlarged ovary with mature oocytes. (F) Mature vitelline cells. Scale bar, 50 μm.

Compared with females recovered from mixed infections, female schistosomes recovered from unisexual infections had no interplay with males *in vivo*, they were stunted in growth and the reproductive system remained fully undeveloped. We also observed that, when newly recovered from infected mice, the development of the reproductive system of virgin females (50 dpi) was retarded compared to that of females (18 dpi) from mixed infections. Fast Blue B failed to stain vitelline cells (Fig 3A), and oogonia in virgin ovaries were smaller than those in females (18 dpi) from mixed infections (Fig 4A, Fig 2A). We investigated whether RBCs can also promote their *in vitro* sexual maturation after females formed pairs with males in culture. To this end, female and male worms (50 dpi) from unisexual infections were cultured together overnight in a basic culture medium to form pairs. When cultured in basic medium, vitelline cells in females could not be stained with Fast Blue B (Table 1). Although development was not apparent by 14 days in medium supplemented with 1% RBCs, Fast Blue B stained-vitelline cells emerged in 35.4% of the paired females when the incubation time was extended to 21 days (Table 1). As shown in Fig 3C and 3D, RBCs stimulated the maturation of vitelline cells, and indeed some of the females produced eggs. These eggs were similar in appearance to wild type eggs of *S*. *japonicum* in human or rodent infections. Under CLSM observation, a large number of mature oocytes was evident in the ovary, and mature vitelline cells were observed at the posterior of the worms (Fig 4E and 4F).

For both unisexual and mixed females cultured *in vitro*, the development of reproductive system to productively release eggs requires both continuous pairing and the presence of erythrocytes, indicating that erythrocytes provide essential nutrients for the reproductive development of females.

### The effect of RBCs fractions on the development of the reproductive system in female *S*. *japonicum in vitro*

Since the intact RBCs promoted the development and maturation of paired pre-adult females cultured *in vitro*, we proceeded to investigate which component of the erythrocyte possessed the promoting activity. WGs and PGs were mainly the membrane part of RBCs, representing the lipid components. The supernatant of lysed RBCs and hemoglobin represented the contents of RBCs. Also, the stimulating effect of disrupted RBCs, the RBCs lysate, was investigated. After incubation *in vitro* for 14 days, only the medium added with intact RBCs promoted the development of the reproductive system of the paired female (18 dpi) (Table 2). Thereafter, we extended the incubation time to 21 days, however, the worms in basic media added with RBC lysate, supernatant of RBCs lysate, WGs, PGs or hemoglobin still did not exhibit any vitelline maturation (data not shown). We speculate from these findings that only the intact RBC provides sufficient nutrition for the development of reproductive organs.

**Table 2:** The percentage of female *S. japonicum* exhibiting vitelline cell development in RPMI-1640 with fractions of RBCs.

RBCs fractions (concentration)	Experiment			Mean
	1	2	3	
Intact RBCs (10 μl/ml)	26.9 (7/26)	20 (3/15)	13.8 (4/29)	20
RBCs lysate (10 μl/ml)	0 (0/25)	0 (0/15)	0 (0/27)	0
Supernatant of RBCs lysate (10 μl/ml)	0 (0/23)	0 (0/15)	3.7 (1/27)	1.5
White ghost (5 μl/ml)	0 (0/22)	0 (0/16)	0 (0/30)	0
Pink ghost (5 μl/ml)	0 (0/25)	0 (0/14)	0 (0/30)	0
Hemoglobin (2 mg/ml)	0 (0/23)	0 (0/15)	0 (0/31)	0

Numbers in brackets represents the number of females positive with Fast Blue B / total number of females paired with males.

### Digested hemoglobin promoted the sexual maturation of pre-adult female *S*. *japonicum in vitro*

*In vivo*, blood is ingested by schistosomes and the hemoglobin from the ingested blood cells flows into the cecum of the parasite. After proteolysis by a battery of schistosome proteases, hemoglobin is degraded to free amino acid and dipeptides [42]. Given that hemoglobin is degraded before absorption, next we analyzed the effect of digested hemoglobin on the development of female reproductive system *in vitro*. We used a neutral protease from *Bacillus subtilis* with a strong ability of protein hydrolysis. This hydrolysis thoroughly fragmented the hemoglobin (S1 Fig). After 14 days of culture in the basic medium supplemented with the hemoglobin hydrolysate, 12.5% of paired female worms were positively stained by Fast Blue B and some even produced eggs; by contrast, reproductive development was not evident in worms cultured with non-protease treated hemoglobin (Table 3).

**Table 3:** The percentage of female *S*. *japonicum* exhibiting vitelline cell development cultured in RPMI-1640 with protein hydrolysate.

Protein hydrolysate	Experiment			Mean
	1	2	3	
None	0 (0/12)	0 (0/10)	0 (0/13)	0
2 mg/ml hemoglobin	0 (0/9)	0 (0/11)	0 (0/7)	0
2 mg/ml hemoglobin hydrolysate	10 (1/10)	8.3 (1/12)	20 (2/10)	12.5
2 mg/ml lactalbumin hydrolysate	10 (1/10)	8.3 (1/12)	30 (3/10)	15.6

Considering that degraded hemoglobin promoted maturation of schistosomes, we hypothesized that the amino acids and dipeptides liberated from hydrolysis of hemoglobin were the main stimulating factors; they are likely to be more rapidly utilized by the worms. Therefore, lactalbumin hydrolysate, a substance replete with free amino acids and dipeptides, was tested for its maturation promoting effect on the female reproductive development. Similar to the function of digested hemoglobin, lactalbumin hydrolysate stimulated female reproductive development. Fast Blue B stained positively the vitelline cells of 15.6% of paired female worms fed with lactalbumin hydrolysate (Table 3).

### Changes in expression of the reproduction-related genes in female worms in the presence of RBCs

**Fig 5:**
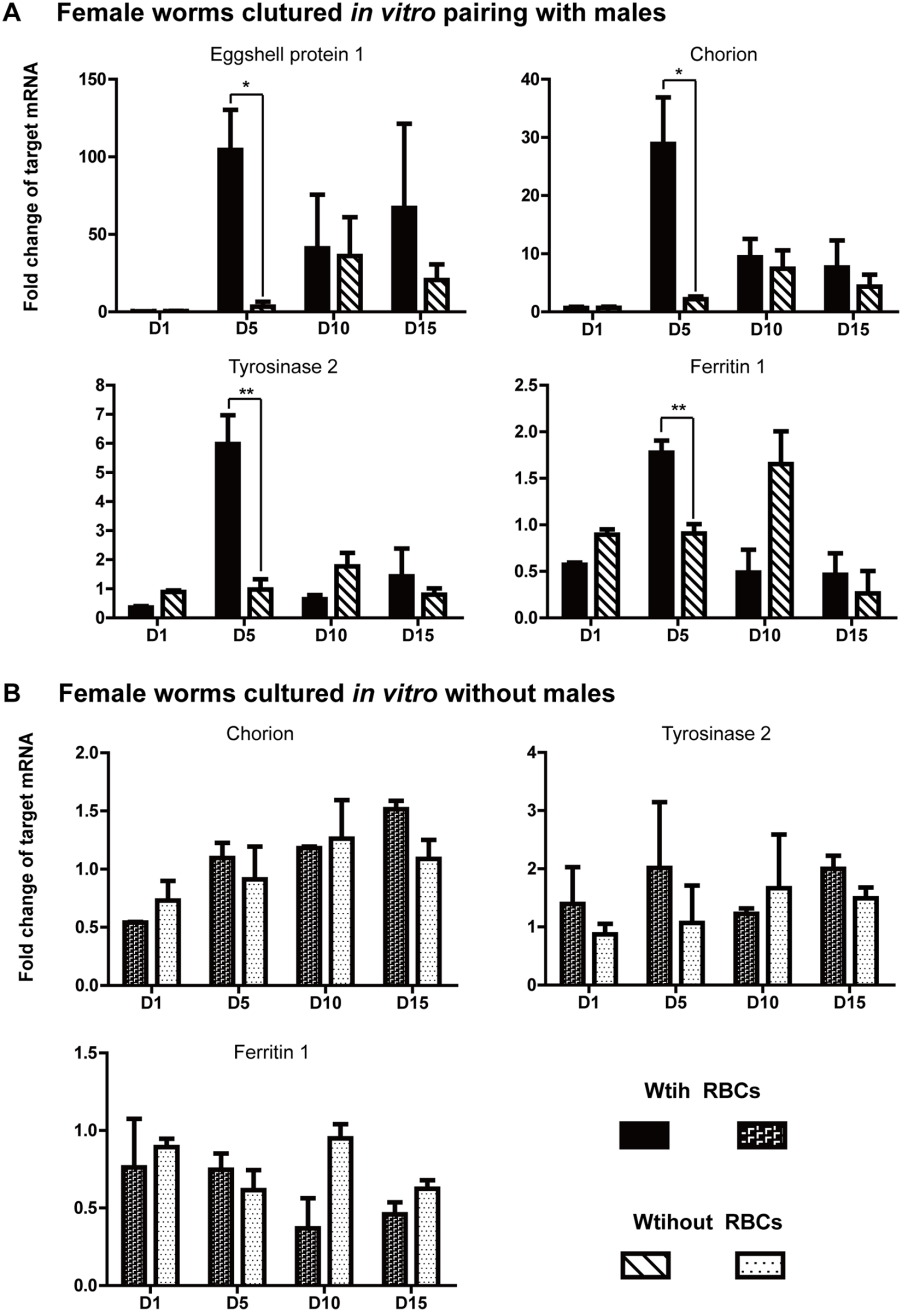
RT-PCR analysis of the expression of the reproduction-related genes of females with and without RBCs. The expression levels of four female reproduction-related genes (eggshell protein 1, chorion, tyrosinase 2 and ferritin 1) were measured by RT-PCR with the PSMD gene as a reference. The fold-change expression was normalized to the expression level of each gene in females at Day 1 without RBC. (A) Fold changes of target genes in females cultured *in vitro* pairing with males during 15 days. (B) Fold changes of target genes in females cultured *in vitro* without males during 15 days.*p<0.05, ** *p*<0.01, three replicates.

Since the RBCs promoted the sexual maturation of the paired pre-adult female worms *in vitro* medium RPMI-1640, we performed qRT-PCR to access the expression patterns of a panel of female reproduction-related genes [38, 39], with or without erythrocytes. These genes were two eggshell proteins (eggshell protein 1, chorion) and two vitelline gland-located genes (tyrosinase 2, ferritin 1). As shown in Fig 5A, the expression of these transcripts increased to the highest levels during the first five days in the presence of RBCs. By contrast, expression levels were significantly (*p*<0.05) lower at this point in the paired females in medium without RBCs. For example, the eggshell protein 1 gene level sharply increased by ~100 fold on day five when co-cultured with RBCs, yet it did not reach to the highest point until day 10 without RBCs. Notably, the expression of these four transcripts in both females with or without RBCs decreased over the subsequent five days. Considering that the female reproductive development is induced by the male schistosome, we then detected the expression changes of these reproduction-related genes in females cultured *in vitro* during 15 days when the male was absent, to investigate the effect of RBCs on gene expression level. In particular, the expression of eggshell protein1 at several culture times could not be detected. The results showed the expression of these three genes, chorion, tyrosinase 2 and ferritin 1, in medium with and without RBCs were in agreement, indicating that they were male-induced. Also, the expression of these genes in female worms isolated from males were identical to those of paired females in medium without RBCs, which suggested gene expression were also RBCs-stimulated (Fig 5B). These findings confirmed that blood cells together with pairing were necessary for expression of these female reproduction-related genes.

## Discussion

Schistosomes uptake host nutrients through the gut and the tegument for growth, development and reproduction [28]. It has long been known that schistosomes ingest RBCs; adult females of *S*. *mansoni* were reported to ingest 330,000 RBCs per hour [23]. In the gut, the hemoglobin is released and then degraded to dipeptides or amino acids by a number of proteases before apparent transport across the gastrodermis into the tissues of the parasite [42]. In addition, iron (Fe) from heme of RBCs is transported to vitelline cells and stored in the eggshell, necessary for the development of the embryo [43]. These findings also suggested that erythrocytes are involved in the nutrition of the sexual development of female schistosomes, though until now without a direct experimental evidence. Here, we confirmed that RBCs promoted the maturation of pre-adult female *S*. *japonicum* cultured *in vitro*. We cultured the pre-adult females in media supplemented with whole serum and observed that the schistosomes remained in copula, although the female reproductive organs remained undeveloped. This indicated that whereas nutrients in serum supported the survival of the pre-adult females they did not facilitate reproductive development. Since blood mainly consists of plasma and red and white blood cells, this finding indicated that the blood cells provided requisite nutrients for the maturation of immature females. Also, we found the expression of gender-related genes of paired female worms were highly stimulated by RBCs, suggesting that female reproductive development was significantly influenced by a nutritional factor. Nevertheless, the *in vitro* culture medium was inferior to growth *in vivo*, since expression of these genes always decreased to low levels after the peak time *in vitro*.

The roles of RBCs or fractions of RBCs in nutrition of schistosomes *in vitro* have been studied. Cheever and Weller [24] reported that the combination of membranes and supernatant promoted growth in culture of schistosomula of *S*. *mansoni* (16 dpi) slightly better than intact RBCs, and far better than membranes alone; they concluded that residual hemoglobin in lysed membranes might be the active component. Lawrence [23] investigated schistosomula of *S*. *mansoni in vitro* and found that PGs were superior to intact RBCs in promoting growth. Schistosomula fed with WGs remained undeveloped even though the hemoglobin, erythrocyte lysate or pressure-liquefied PGs was added subsequently. It was presumed that the remaining hemoglobin in PGs was more accessible to schistosomula. By contrast, in the present study, cultured pre-adult *S*. *japonicum* fed with intact RBCs rather than the fractions exhibited direct reproductive development, and the emergence in culture of noteworthy changes in morphology of the gonads. We postulate two explanations for this outcome. First, intact RBCs settle by gravity to the floor of the dish where the worms usually are located, which provided a microenvironment with a locally high concentration of nutrients. In contrast, equivalent amounts of fractions, including RBC lysate, lysate supernatant or hemoglobin, were dissolved in the culture medium, thus the concentration of these nutrients around worms was lower. However, when we tried to increase the supplies of these fractions in the media, the pairing activity of worms was negatively impacted (data not shown). Although most PGs settled to the bottom of the dish, the content of the PGs was markedly less than that of intact RBCs. Intact RBCs therefore provided more nutrients in smaller volume. Second, in culture medium, the worms were likely exposed to heme and reactive oxygen species released from the lysate of RBCs. *In vivo*, these harmful substances are probably detoxified rapidly in the gut [44]. By contrast, the activities of worms *in vitro* might be inhibited by these xenobiotics. To verify this notion, when we cultured pre-adult worms with intact RBCs and lysate of RBCs, the reproductive development was not affected (data not shown). This suggested that the superiority of intact RBCs to its fractions was principally related to the nutrient rich microenvironment it established. Moreover, since PGs delivered better growth of schistosomula whereas intact RBCs delivered superior reproductive development, nutritional requirements may vary among the developmental stages of the schistosome.

Hemoglobin contributes >95% of the dry mass of the erythrocyte. Host hemoglobin is apparently essential for schistosome nutrition [22]. A battery of proteases participates in the degradation of hemoglobin to diffusible amino acids and peptides [42, 45]. However, a stimulatory effect of hemoglobin on the development of the reproductive system in schistosomes has never been confirmed *in vitro*. The present findings demonstrated that the worms fed on the protease-digested hemoglobin rather than untreated-hemoglobin exhibited clear reproductive development. It appears likely that the hemoglobin was more available in medium after hydrolysis by neutral protease. Considering that schistosomes take up glucose, amino acids and other nutrients through their tegumental surface in addition to the gut [28], we predicted that the free amino acids from hemoglobin hydrolysate were transported through both tegument and gut, which satisfied the high nutrient demand of reproductive development. Development of vitelline cells was reported in *S*. *mansoni* females from unisexual infections pairing with males after five days in culture, in a mixture of 50% human serum and 50% Earle’s balanced salt solution containing 6.5 mg/ml lactalbumin hydrolysate [46]. In the present study, reproductive development was not observed in paired pre-adult females from mixed infections cultured in the RPMI-1640 with 50% serum. When 2 mg/ml lactalbumin hydrolysate was included, the maturation of vitellaria and ovary proceeded. This supported the notion that sources of nitrogen are necessary for sexual development of the female schistosome and the free amino acids and peptides were more readily absorbed than intact proteins/large polypeptides by worms *in vitro*.

The females (18 dpi) in medium with RBCs did not exhibit detectable changes in their vitellaria during the first seven days *in vitro*, whereas more than 20% of females matured over the subsequent seven days. For the females (50 dpi), notably rapid development of vitelline cells was observed between day 14 to 21. According to Erasmus’ categorization [47], vitelline cells are classified into four stages. The unisexual females only contained the stage 1 cells, while the vitelline cells were not stainable by Fast Blue B until they reached stage 3 or stage 4 when the typical vitelline droplets were formed. It is possible that vitelline cells of females from unisexual infections developed to stage 2 within the first 14 days *in vitro*, and by the last seven days many vitelline cells reached stage 3 and stage 4. This also could explain the shorter incubation time required for females from mixed infections than worms from unisexual infections to mature, for the vitelline cells of females from mixed infections had already been induced to develop by males *in vivo*. Since these immature females matured and produced eggs *in vitro*, we then investigated the quality of these laid eggs. Although the shape and appearance of the eggs was apparently normal, as established using staining with Fast Blue B, the egg-shell was not smooth when viewed by CLSM (S2 Fig). Furthermore, we did not observe any miracidia hatching from the eggs. This suggested that other host factors, not present in our culture media, are required for normal development of the egg. This warrants further investigation.

Understanding the molecular and biochemical regulation of sexual development and maturation occurring in schistosomes is valuable since disruption of these processes may provide leads for intervention, including the vaccines and drugs for controlling schistosomiasis. However, the physiology of male-induced female reproduction is not well understood [6]. This is likely due in part because, at present, the developmental requirements of female maturation can only be achieved *in vivo*. Nonetheless, *in vitro* culture can be informative in investigations of the reproductive biology of this pathogen. Medium 841 is a rich medium that can support the development of *S*. *japonicum* from cercariae to adults. However, it takes 80 days before the cultured blood flukes produce eggs; and the eggs produced are abnormal. Therefore, the *in vitro* development is much slower than that *in vivo*. Here, when cultured in basic medium with intact RBCs or hemoglobin hydrolysate, the pre-adult female *S*. *japonicum* worms presented detectable vitelline maturation in one week and laid eggs in two weeks. This less complex medium and shorter culture time represent a technological advance that can be expected to enhance research on the sexual biology and reproduction of schistosomes.

## Conclusions

Besides pairing with male, the reproductive development of female *S*. *japonicum* requires nutritional support. Our findings confirmed it is possible to culture immature pre-adult worms to egg producing worms in the presence of either red blood cells or hemaglobin hydrolysate or lactalbumin hydrolysate. The bovine hemoglobin hydrolysate promoted female sexual maturation instead of intact hemoglobin, suggesting that free amino acid and dipeptides were more efficient for stimulating the reproductive development than complete proteins in culture medium. In addition, RBCs significantly promoted the expression of reproduction-related genes in paired female worms cultured *in vitro*. Yet, the *in vitro* developed female schistosomes were not fully matured, for their eggs were still infertile. Understanding the nutritional requirements for schistosome reproductive development can be expected to aid optimization of *in vitro* culture methods for these parasites, and provide insights for innovative ways for treatment of schistosomiasis.

## Supporting Information

S1 Fig SDS-PAGE analysis of the proteolysis of the bovine hemoglobin. Lane M, protein markers; lane 1, bovine hemoglobin; lane 2, bovine hemoglobin hydrolysate catalyzed by neutral protease.
(TIF)

S2 Fig CLSM images of eggs in female *S*. *japonicum* developed *in vitro*. (A) Egg formation in the ootype. (B) Accumulated eggs near the genital pore.
(TIF)

S1 Table Raw data from the RT-PCR. (XLSX)

S2 Table Summary of the statistical analysis results. (XLSX)
