# Supplementary figures and images for: Intake of Erythrocytes Required for Reproductive Development of Female Schistosoma japonicum
Source: PLoS One. 2015 May 15;10(5):e0126822. doi: 10.1371/journal.pone.0126822 (PMC4433235; doi:10.1371/journal.pone.0126822)

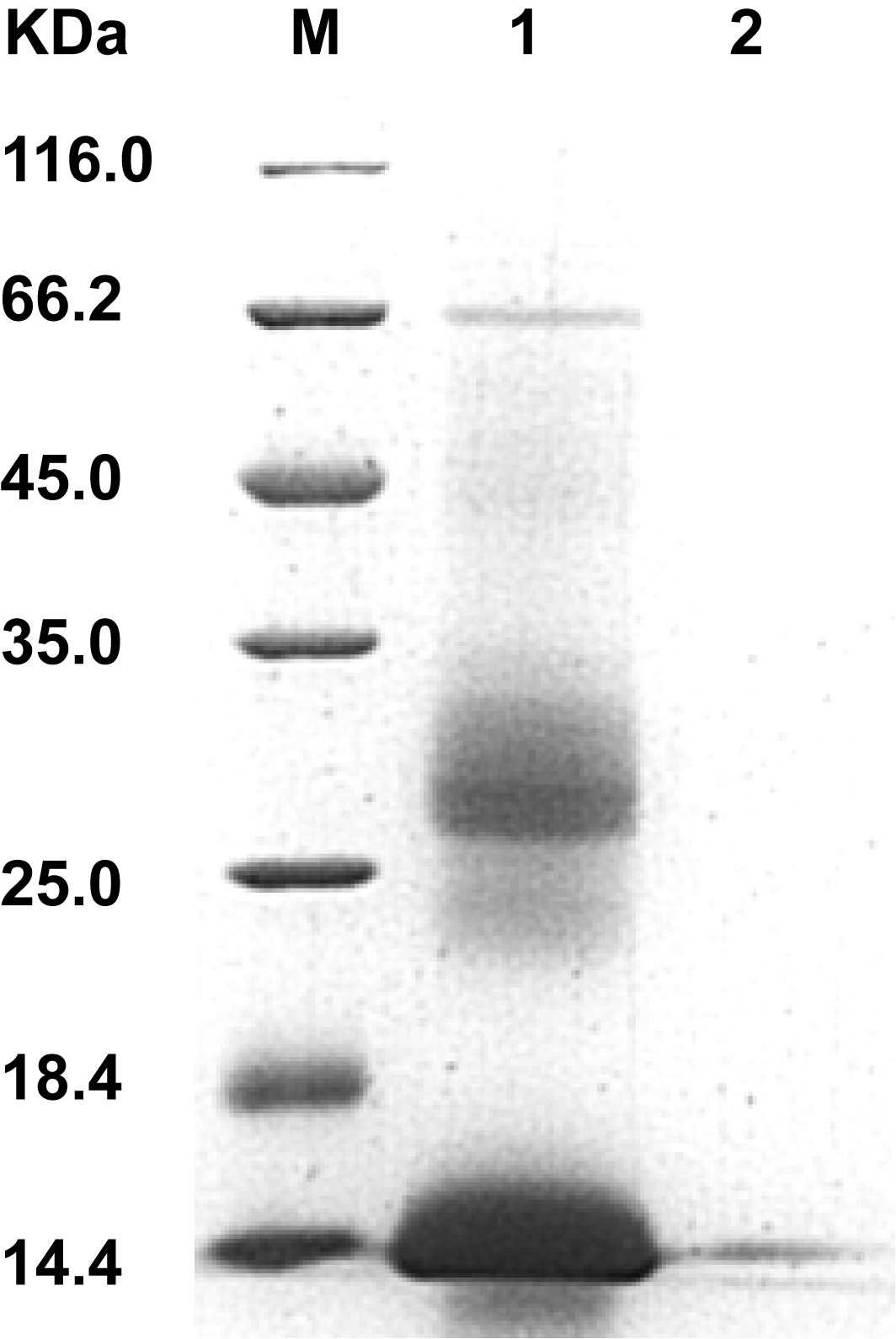

Supplement: S1 Fig — Lane M, protein markers; lane 1, bovine hemoglobin; lane 2, bovine hemoglobin hydrolysate catalyzed by neutral protease. (TIF) [file pone.0126822.s001.tif]

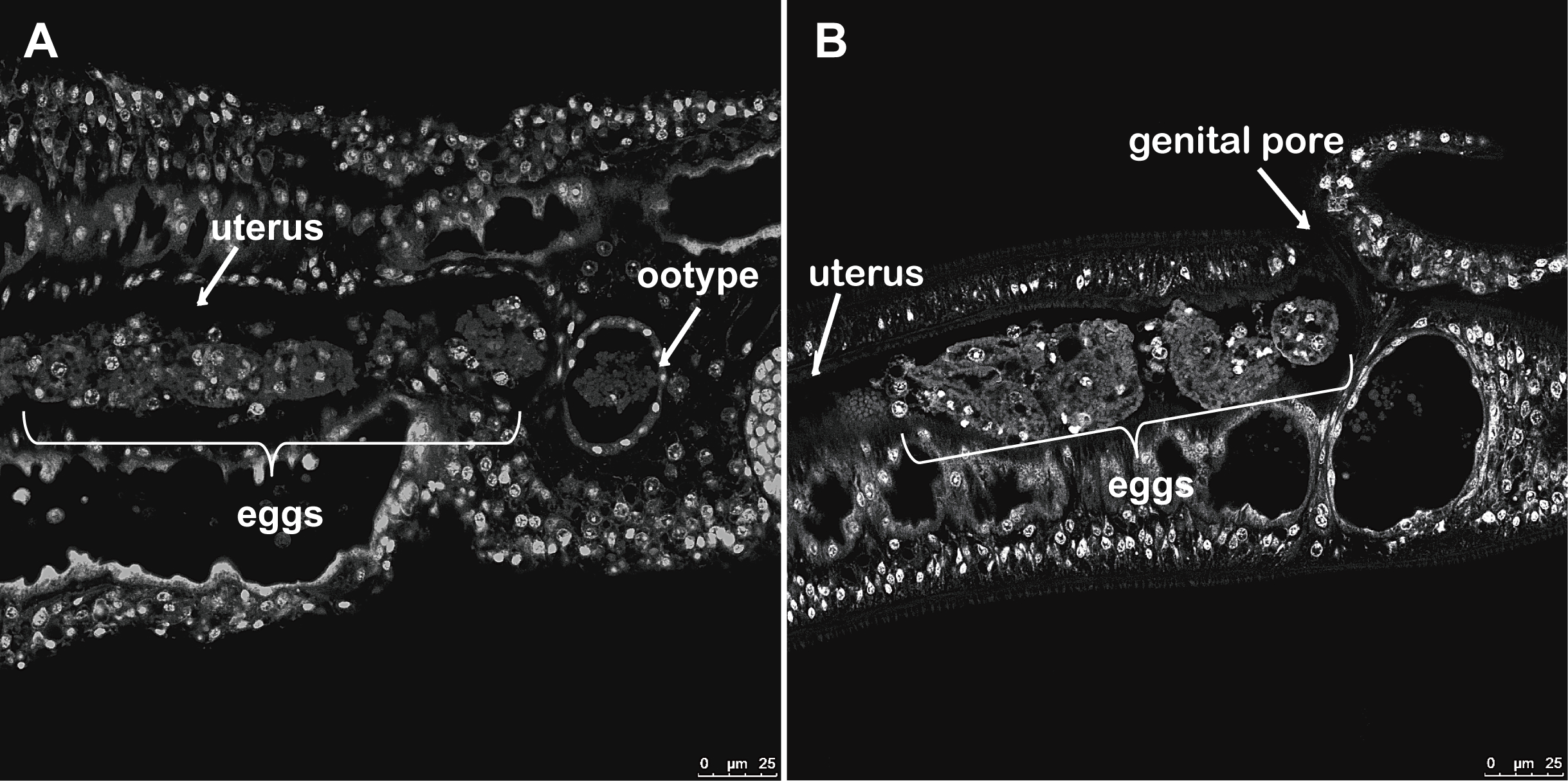

Supplement: S2 Fig — (A) Egg formation in the ootype. (B) Accumulated eggs near the genital pore. (TIF) [file pone.0126822.s002.tif]
